# Crystal structure of (3*E*)-5-nitro-3-(2-phenyl­hydrazinyl­idene)-1*H*-indol-2(3*H*)-one

**DOI:** 10.1107/S2056989016020375

**Published:** 2017-01-13

**Authors:** Jecika Maciel Velasques, Vanessa Carratu Gervini, Adaílton João Bortoluzzi, Renan Lira de Farias, Adriano Bof de Oliveira

**Affiliations:** a Universidade Federal do Rio Grande (FURG), Escola de Química e Alimentos, Rio Grande, Brazil; b Universidade Federal de Santa Catarina (UFSC), Departamento de Química, Florianópolis, Brazil; c Universidade Estadual Paulista (UNESP), Instituto de Química, Araraquara, Brazil; d Universidade Federal de Sergipe (UFS), Departamento de Química, São Cristóvão, Brazil

**Keywords:** crystal structure, isatin–hydrazone derivative, two-dimensional hydrogen-bonded network, Hirshfeld surface calculation, *in silico* evaluation

## Abstract

The reaction between 5-nitro­isatin and phenyl­hydrazine in acidic ethanol yields the title compound, C_14_H_10_N_4_O_3_, whose mol­ecular structure deviates slightly from a planar geometry (r.m.s. deviation = 0.065 Å for the mean plane through all non-H atoms). An intra­molecular N—H⋯O hydrogen bond is present, forming a ring of graph-set motif *S*(6). In the crystal, mol­ecules are linked by N—H⋯O and C—H⋯O hydrogen-bonding inter­actions into a two-dimensional network along (120), and rings of graph-set motif *R*_2_^2^(8), *R*_2_^2^(26) and *R*_4_^4^(32) are observed. Additionally, a Hirshfeld surface analysis suggests that the mol­ecules are stacked along [100] through C=O⋯*Cg* inter­actions and indicates that the most important contributions for the crystal structure are O⋯H (28.5%) and H⋯H (26.7%) inter­actions. An *in silico* evaluation of the title compound with the DHFR enzyme (di­hydro­folate reductase) was performed. The isatin–hydrazone derivative and the active site of the selected enzyme show N—H⋯O(*ASP29*), N—H⋯O(*ILE96*) and *Cg*⋯*Cg*(*PHE33*) inter­actions.

## Abstract 2

The mol­ecule of 5-nitro­isatin-3-phenyl­hydrazone deviates slightly from a planar geometry. In the crystal, mol­ecules are linked by hydrogen bonding into a two-dimensional polymer along (120), forming rings of graph-set motifs 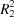
(8), 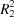
(26), 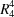
(32) and *S*(6). In addition, mol­ecules are stacked along the [100] through C=O⋯*Cg* inter­actions, as suggested by the Hirshfeld surface, which also indicates that the most important contributions for the crystal structure cohesion are O⋯H (28.5%) and H⋯H (26.7%) inter­actions. An *in silico* evaluation of the title compound with the di­hydro­folate reductase enzyme was performed and N—H⋯O and *Cg*⋯*Cg* inter­actions were found.

## Chemical context   

The first reports on isatin and the synthesis of isatin derivatives were published independently in Germany and France over 170 years ago (Erdmann, 1841*a* ▸,*b* ▸; Laurent, 1841 ▸). After the 19th Century, isatin chemistry changed rapidly into a major group of compounds with a wide range of applications in different scientific disciplines, with special attention to medicinal chemistry. For example, the synthesis, *in silico* evaluation and *in vitro* inhibition of Chikungunya virus replication by an isatin–thio­semicarbazone derivative was performed recently (Mishra *et al.*, 2016 ▸). Other isatin derivatives synthesized in the 1950s (Campaigne & Archer, 1952 ▸) had their pharmacological properties *in vitro* successfully tested against Cruzain, Falcipain-2 and Rhodesian in the 2000s (Chiyanzu *et al.*, 2003 ▸), and the crystal structure of one of the derivatives was determined by X-ray diffraction in the 2010s (Pederzolli *et al.*, 2011 ▸). The crystal structure determination of isatin-based mol­ecules is an intensive research field, especially in medicinal chemistry. As part of our studies in this area, we now describe the synthesis and structure of the title compound, (I).

## Structural commentary   

**Figure 1:**
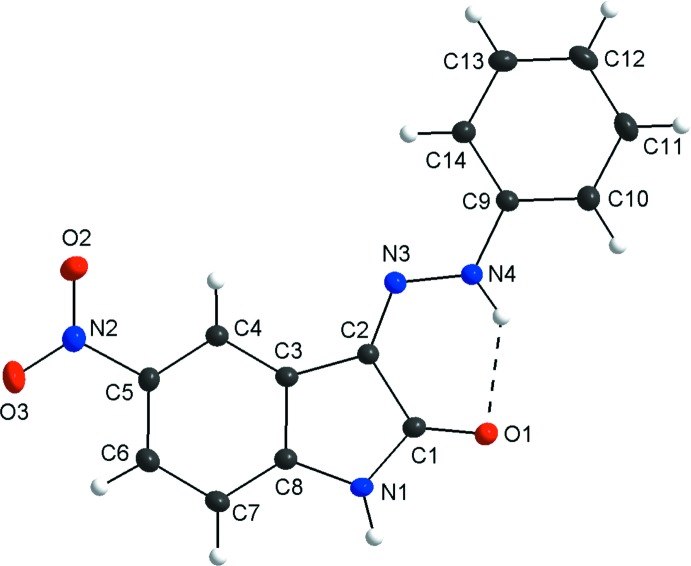
The mol­ecular structure of the title compound, showing displacement ellipsoids drawn at the 50% probability level. The intra­molecular hydrogen bond is shown as a dashed line.

For the title compound, the mol­ecular structure matches the asymmetric unit and one intra­molecular N4—H5⋯O1 inter­action of graph-set *S*(6) is observed (Fig. 1 ▸). The mol­ecule is nearly planar with an r.m.s. deviation from the mean plane of the non–H atoms of 0.065 Å and a maximum deviation of 0.1907 (9) Å for atom O2 of the nitro group. The dihedral angle between the indole unit and the phenyl ring is 0.9 (4)°. The plane through the nitro group is rotated by 6.21 (6)° with respect to the indole ring.
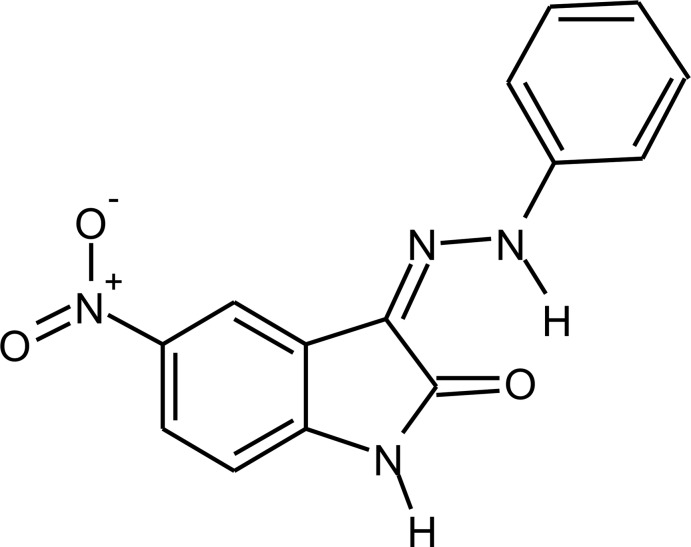


## Supra­molecular features   

**Figure 2:**
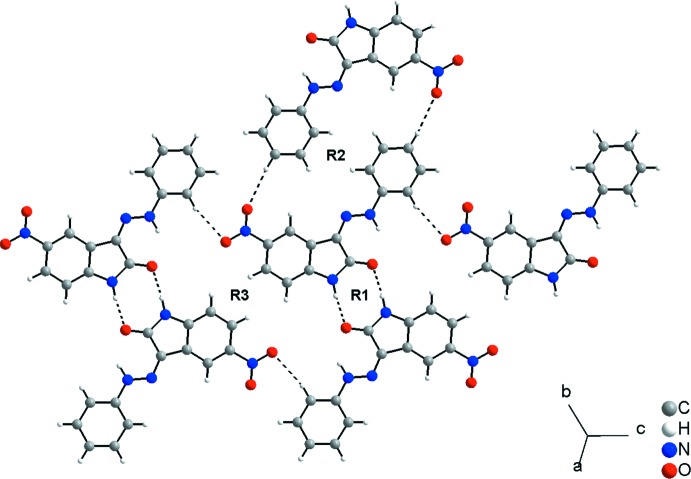
A packing diagram of the title compound, showing the N—H⋯O and C—H⋯O inter­actions (dashed lines) connecting the mol­ecules into a two-dimensional network in the (120) plane. The graph-set motifs for the crystal packing are: R1 = 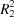
(8), R2 = 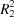
(26) and R3 = 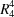
(32).

**Figure 3:**
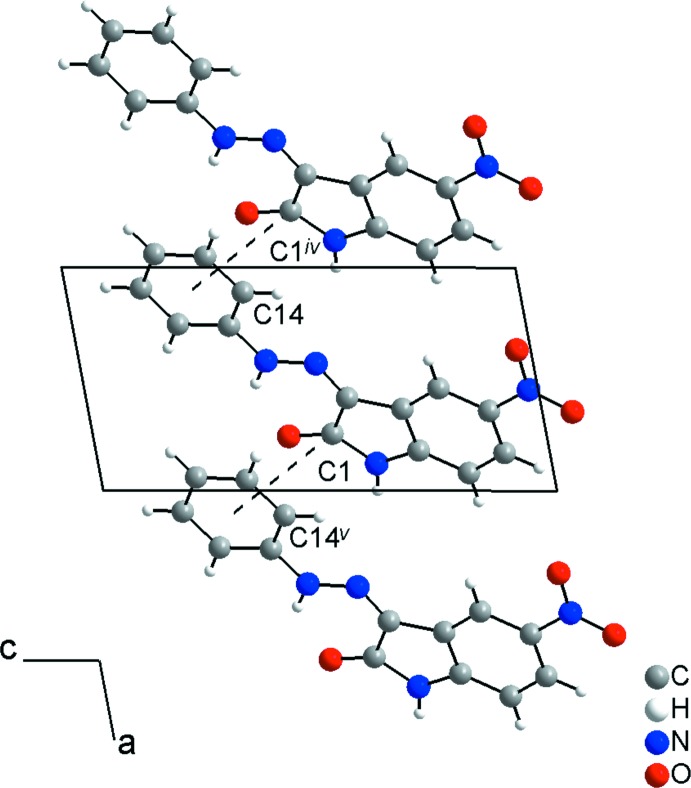
A packing diagram of the title compound showning the C⋯*Cg* inter­actions (as dashed lines) building a chain along [100]. [Symmetry codes: (iv) *x* − 1, *y*, *z*; (v) *x* + 1, *y*, *z*.]

In the crystal, the mol­ecules are connected by centrosymmetric pairs of N1—H1⋯O1^i^ inter­actions (Table 1 ▸) into dimers with graph-set motif 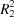
(8). In addition, C10—H6⋯O3^ii^ and C12—H8⋯O2^iii^ inter­actions complete a two-dimensional hydrogen-bonded network with rings of graph-set motif 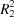
(26) and 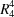
(32) (Fig. 2 ▸, Table 1 ▸). As suggested by Hirshfeld surface analysis, the dimensionality of the structure increases to three-dimensional through the C=O⋯*Cg* inter­actions [C1⋯*Cg* = 3.5427 (7) Å, O1⋯*Cg* = 3.2004 (7) Å; *Cg* is the centroid of the C9–C14 ring], building a chain along [100] (Fig. 3 ▸). The separation between the C1 and C14 atoms of adjacent mol­ecules in the chain is 3.1744 (11) Å, which is shorter than the sum of the van der Waals radii for carbon atoms (Bondi, 1964 ▸; Rowland & Taylor, 1996 ▸).

## Hirshfeld surface analysis   

**Figure 4:**
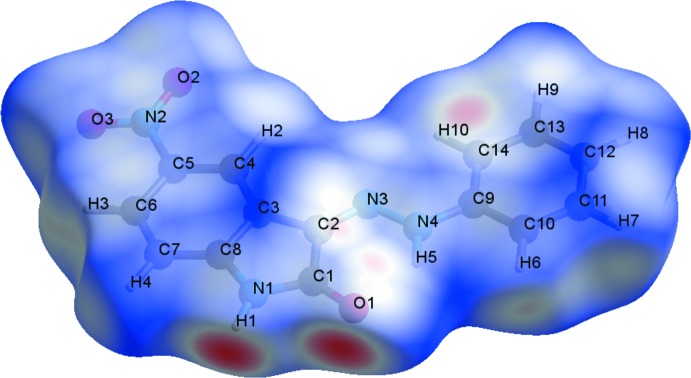
A Hirshfeld surface graphical representation (*d*_norm_) for the title compound. The surface is drawn with transparency and all atoms are labelled. The surface regions with strongest inter­molecular inter­actions for atoms H1, O1 and C14 are shown in magenta.

**Figure 6:**
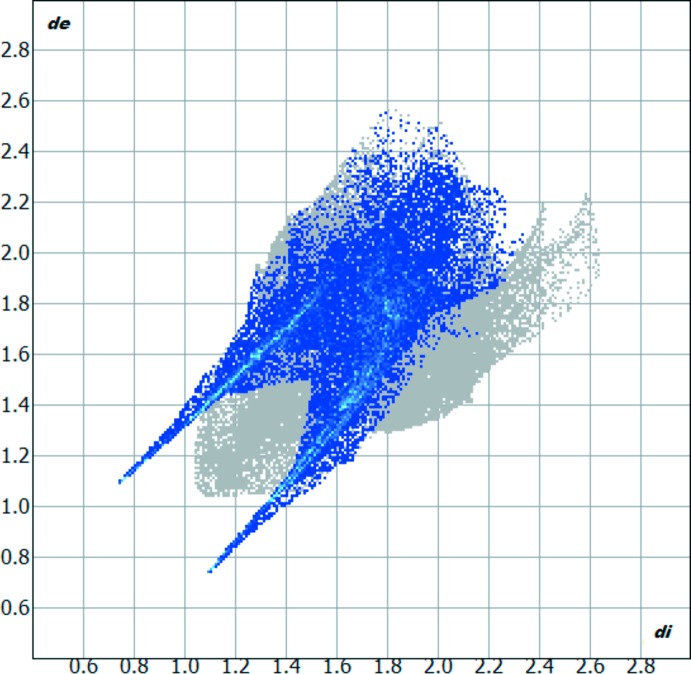
Hirshfeld surface fingerprint two-dimensional plot for the 5-nitro­isatin-3-phenyl­hydrazone crystal structure showing the O⋯H contacts in detail (cyan dots). The O⋯H contribution for the crystal packing amounts to 28.5%, being the most important inter­molecular connection. The *d*_e_ (*y* axis) and *d*_i_ (*x* axis) values are the closest external and inter­nal distances [in Å] from given points on the Hirshfeld surface.

The Hirshfeld surface analysis of the crystal structure indicates that the contribution of O⋯H inter­molecular inter­actions to the crystal packing amounts to 28.5% and the H⋯H inter­actions amount to 26.7%. Other important inter­molecular contacts for the cohesion of the structure are (in %): H⋯C = 17.7, H⋯N = 8.9, C⋯O = 8.2, C⋯C = 5.5 and C⋯N = 3.3. The Hirshfeld surface graphical representation with transparency and labelled atoms (Figs. 4 ▸ and 5 ▸) indicates, in magenta, the locations of the strongest inter­molecular contacts. The H1, H8, O1 and O2 atoms are the most important for the inter­molecular hydrogen bonding, while the C1 and C14 atoms are the most important for C⋯C inter­actions. The O⋯H contribution to the crystal packing is shown as a Hirshfeld surface fingerprint two-dimensional plot with cyan dots (Wolff *et al.*, 2012 ▸). The *d*_e_ (*y* axis) and *d*_i_ (*x* axis) values are the closest external and inter­nal distances (in Å) from given points on the Hirshfeld surface (Fig. 6 ▸). The magenta colour on graphical representations of the Hirshfeld surface matches the N1—H1⋯O1^*i*^, C10—H6⋯O3^*ii*^ and C12—H8⋯O2^*iii*^ inter­actions described above. In the same way, the C⋯*Cg* inter­actions can be seen more clearly on the C1=O1 and C14 atoms.

## Mol­ecular docking evaluation   

**Figure 7:**
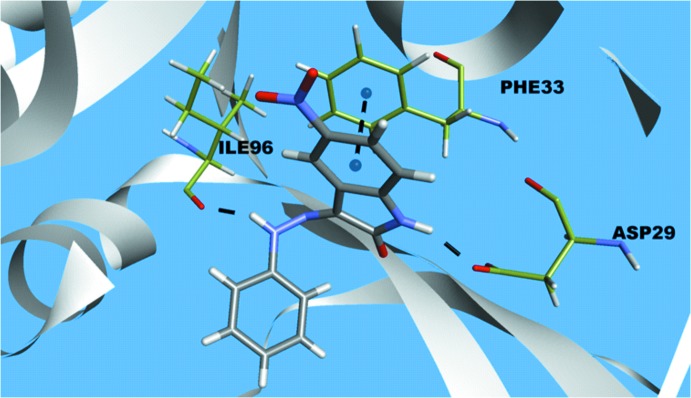
Inter­molecular inter­actions between the title compound and the di­hydro­folate reductase enzyme. The inter­actions are shown as dashed lines and the figure is simplified for clarity.

Finally, for a lock-and-key supra­molecular analysis, a mol­ecular docking evaluation between the title compound and the DHFR enzyme (di­hydro­folate reductase) was carried out. Initially, the semi-empirical equilibrium energy of the small mol­ecule was obtained using the PM6 Hamiltonian, but the experimental bond lengths were conserved. The calculated parameters were: heat of formation = 149.41 kJ mol^−1^, gradient normal = 0.763, HOMO = −8.96 eV, LUMO =-1.66 eV and energy gap = 7.30 eV. The target prediction for 5-nitro­isatin-3-phenyl­hydrazone was calculated with the *SwissTargetPrediction* webserver based on the bioisosteric similarity to the isatin entity (Gfeller *et al.*, 2013 ▸). As result of this screening, the title compound showed a promising theoretical structure–activity relationship to kinase proteins sites. The Frequency Target Class for kinases amounts to 44%, while the second best result for phosphatases amounts to 13%. The inter­actions with enzymes are important features for biologic­ally active mol­ecules, *e.g.* inhibition of tumor cell proliferation, activation of cell apoptosis mechanisms and blocking of bacterial membrane synthesis. Based on a search for a biological target with pharmacological background, the di­hydro­folate reductase was selected for the *in silico* evaluation (Chen, 2015 ▸; Dias *et al.*, 2014 ▸; Verdonk *et al.*, 2003 ▸), biological target code: DHFR (Protein Data Bank ID: 4KM0; Wei *et al.*, 2005 ▸). The isatin–hydrazone derivative and the active site of the selected enzyme matches and the structure–activity relationship can be assumed by the following observed inter­molecular inter­actions: N1—H1⋯O(*ASP29*) (1.928 Å), N4—H5⋯O(*ILE96*) (1.925 Å) and *Cg*⋯*Cg*(*PHE33*) (3.567 Å) (Fig. 7 ▸).

## Comparison with a related structure   

A recently published article (Bittencourt *et al.*, 2016 ▸) reports the structure of (3*E*)-5-nitro-3-(2-phenyl­hydrazinyl­idene)-1*H*-indol-2(3*H*)-one, which may be compared with that of the title compound. The mol­ecular structure deviates slightly from the ideal planar geometry and the C⋯C contacts between the planes are observed. The mol­ecules are linked by N—H⋯O and C—H⋯Cl inter­actions into a two-dimensional hydrogen-bonded polymer, a quite similar structure to the title compound. The *in silico* evaluation of 5-chloro­isatin-phenyl­hydrazone, a mol­ecule with similar crystal packing to the title compound, with and the DNA topoisomerase IIα enzyme was performed and the global free energy of −26.59 kJ mol^−1^ was found. The evaluation agrees with the literature data for mol­ecular docking and cytotoxic activity of hydrazone derivatives against breast cancer cells (Dandawate *et al.*, 2012 ▸) and supports research on the structural determination of other isatin-based mol­ecules. The title compound is commercially available, but its structural analysis by X–ray single crystal diffraction, Hirshfeld surface calculation and mol­ecular docking evaluation are presented in this work for the first time.

## Synthesis and crystallization   

All starting materials are commercially available and were used without further purification. The synthesis of the title compound was adapted from a procedure reported previously (Fonseca *et al.*, 2011 ▸). The glacial acetic acid-catalysed reaction of 5-nitro­isatin (2.6 mmol) and phenyl­hydrazine (2.6 mmol) in ethanol (40 mL) was refluxed for 4 h. After cooling and filtering, an irregular solid was isolated. Single crystals suitable for X-ray diffraction were obtained from a DMF/methanol solution (1:1 *v*/*v*) on slow evaporation of the solvent.

## Refinement   

Crystal data, data collection and structure refinement details are summarized in Table 2 ▸. Hydrogen atoms were located in a difference Fourier map, but were positioned with idealized geometry and refined isotropically using a riding model, with *U*_iso_(H) = 1.2*U*_eq_(C, N), and with C—H = 0.95 Å and N—H = 0.88 Å.

## Supplementary Material

Crystal structure: contains datablock(s) I, publication_text. DOI: 10.1107/S2056989016020375/rz5203sup1.cif

Structure factors: contains datablock(s) I. DOI: 10.1107/S2056989016020375/rz5203Isup2.hkl

SwissTargetPrediction Report for 5-nitroisatin-3-phenylhydrazone. DOI: 10.1107/S2056989016020375/rz5203sup3.pdf

Supporting information file. DOI: 10.1107/S2056989016020375/rz5203Isup4.cml

CCDC reference: 1524161

Additional supporting information:  crystallographic information; 3D view; checkCIF report

## Figures and Tables

**Figure 5:**
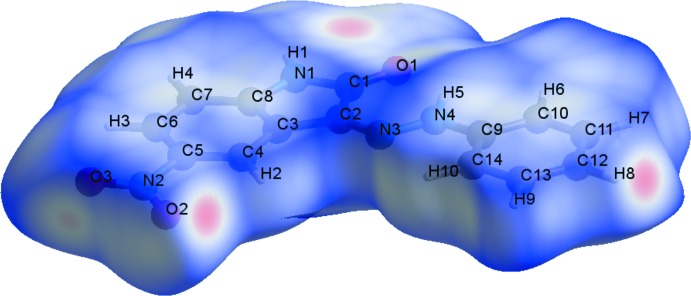
A Hirshfeld surface graphical representation (*d*_norm_) for the title compound. The surface is drawn with transparency and all atoms are labelled. The surface regions with strongest inter­molecular inter­actions for atoms H8, O2 and C1 are shown in magenta.

**Table 1:** Hydrogen-bond geometry (Å, °)

*D*—H⋯*A*	*D*—H	H⋯*A*	*D*⋯*A*	*D*—H⋯*A*
N4—H5⋯O1	0.88	2.03	2.7479 (10)	137
N1—H1⋯O1^i^	0.88	1.96	2.8310 (10)	171
C10—H6⋯O3^ii^	0.95	2.63	3.5542 (13)	166
C12—H8⋯O2^iii^	0.95	2.47	3.3943 (13)	163

Symmetry codes: (i) 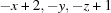; (ii) 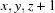; (iii) 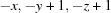.

**Table 2:** Experimental details

Crystal data	
Chemical formula	C_14_H_10_N_4_O_3_
*M*_r_	282.26
Crystal system, space group	Triclinic, *P* 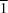
Temperature (K)	200
*a*, *b*, *c* (Å)	5.7504 (4), 9.7190 (6), 12.1976 (7)
α, β, γ (°)	111.196 (2), 96.759 (2), 98.497 (2)
*V* (Å^3^)	617.69 (7)
*Z*	2
Radiation type	Mo *K*α
μ (mm^−1^)	0.11
Crystal size (mm)	0.48 × 0.16 × 0.10

Data collection	
Diffractometer	Bruker APEXII CCD area detector
Absorption correction	Multi-scan (*SADABS*; Bruker, 2013 ▸)
*T*_min_, *T*_max_	0.949, 0.989
No. of measured, independent and observed [*I* > 2σ(*I*)] reflections	11325, 3971, 3281
*R*_int_	0.017
(sin θ/λ)_max_ (Å^−1^)	0.726

Refinement	
*R*[*F* ^2^ > 2σ(*F* ^2^)], *wR*(*F* ^2^), *S*	0.039, 0.117, 1.03
No. of reflections	3971
No. of parameters	190
H-atom treatment	H-atom parameters constrained
Δρ_max_, Δρ_min_ (e Å^−3^)	0.37, −0.26

Computer programs: *APEX2* and *SAINT* (Bruker, 2013 ▸), *SHELXS97* and *SHELXL97* (Sheldrick, 2008 ▸), *DIAMOND* (Brandenburg, 2006 ▸), *GOLD* (Verdonk *et al.*, 2003 ▸), *Crystal Explorer* (Wolff, *et al.*, 2012 ▸), *publCIF* (Westrip, 2010 ▸) and *enCIFer* (Allen *et al.*, 2004 ▸).

## supplementary crystallographic information

### Crystal data

**Table:**

C_14_H_10_N_4_O_3_	*Z* = 2
*M**_r_* = 282.26	*F*(000) = 292
Triclinic, *P*1	*D*_x_ = 1.518 Mg m^−^^3^
*a* = 5.7504 (4) Å	Mo *K*α radiation, λ = 0.71073 Å
*b* = 9.7190 (6) Å	Cell parameters from 2154 reflections
*c* = 12.1976 (7) Å	θ = 2.3–31.0°
α = 111.196 (2)°	µ = 0.11 mm^−^^1^
β = 96.759 (2)°	*T* = 200 K
γ = 98.497 (2)°	Prism, yellow
*V* = 617.69 (7) Å^3^	0.48 × 0.16 × 0.10 mm

### Data collection

**Table:**

Bruker APEXII CCD area detector diffractometer	3971 independent reflections
Radiation source: fine-focus sealed tube, Bruker APEX2 CCD	3281 reflections with *I* > 2σ(*I*)
Graphite monochromator	*R*_int_ = 0.017
φ and ω scans	θ_max_ = 31.1°, θ_min_ = 1.8°
Absorption correction: multi-scan (SADABS; Bruker, 2013)	*h* = −8→8
*T*_min_ = 0.949, *T*_max_ = 0.989	*k* = −14→14
11325 measured reflections	*l* = −17→17

### Refinement

**Table:**

Refinement on *F*^2^	Primary atom site location: structure-invariant direct methods
Least-squares matrix: full	Secondary atom site location: difference Fourier map
*R*[*F*^2^ > 2σ(*F*^2^)] = 0.039	Hydrogen site location: inferred from neighbouring sites
*wR*(*F*^2^) = 0.117	H-atom parameters constrained
*S* = 1.03	*w* = 1/[σ^2^(*F*_o_^2^) + (0.0693*P*)^2^ + 0.1171*P*] where *P* = (*F*_o_^2^ + 2*F*_c_^2^)/3
3971 reflections	(Δ/σ)_max_ < 0.001
190 parameters	Δρ_max_ = 0.37 e Å^−^^3^
0 restraints	Δρ_min_ = −0.26 e Å^−^^3^

### Special details

**Table:**

Geometry. All esds (except the esd in the dihedral angle between two l.s. planes) are estimated using the full covariance matrix. The cell esds are taken into account individually in the estimation of esds in distances, angles and torsion angles; correlations between esds in cell parameters are only used when they are defined by crystal symmetry. An approximate (isotropic) treatment of cell esds is used for estimating esds involving l.s. planes.
Refinement. Refinement of F^2^ against ALL reflections. The weighted R-factor wR and goodness of fit S are based on F^2^, conventional R-factors R are based on F, with F set to zero for negative F^2^. The threshold expression of F^2^ > 2sigma(F^2^) is used only for calculating R-factors(gt) etc. and is not relevant to the choice of reflections for refinement. R-factors based on F^2^ are statistically about twice as large as those based on F, and R- factors based on ALL data will be even larger.

### Fractional atomic coordinates and isotropic or equivalent isotropic displacement parameters (Å^2^)

**Table:**

	*x*	*y*	*z*	*U*_iso_*/*U*_eq_	
C1	0.74444 (16)	0.10997 (10)	0.47144 (8)	0.01734 (17)	
C2	0.58388 (16)	0.19030 (10)	0.42434 (8)	0.01692 (17)	
C3	0.63293 (16)	0.17378 (10)	0.30690 (8)	0.01701 (17)	
C4	0.53990 (17)	0.22036 (10)	0.21904 (8)	0.01863 (18)	
H2	0.4130	0.2737	0.2283	0.022*	
C5	0.64117 (17)	0.18525 (11)	0.11683 (8)	0.02053 (19)	
C6	0.8299 (2)	0.10968 (12)	0.10014 (9)	0.0255 (2)	
H3	0.8968	0.0921	0.0299	0.031*	
C7	0.92000 (19)	0.06009 (12)	0.18685 (9)	0.0240 (2)	
H4	1.0462	0.0062	0.1767	0.029*	
C8	0.81885 (16)	0.09218 (10)	0.28844 (8)	0.01844 (17)	
C9	0.26077 (16)	0.34671 (10)	0.65458 (8)	0.01776 (17)	
C10	0.25473 (19)	0.34151 (12)	0.76695 (9)	0.0244 (2)	
H6	0.3603	0.2920	0.7976	0.029*	
C11	0.0939 (2)	0.40895 (13)	0.83359 (9)	0.0296 (2)	
H7	0.0883	0.4047	0.9099	0.036*	
C12	−0.0597 (2)	0.48282 (13)	0.78973 (10)	0.0284 (2)	
H8	−0.1711	0.5280	0.8354	0.034*	
C13	−0.04899 (19)	0.49000 (11)	0.67893 (9)	0.0248 (2)	
H9	−0.1521	0.5418	0.6494	0.030*	
C14	0.11070 (18)	0.42243 (11)	0.61027 (9)	0.02091 (19)	
H10	0.1173	0.4278	0.5344	0.025*	
N1	0.87794 (14)	0.05403 (9)	0.38620 (7)	0.01994 (17)	
H1	0.9867	0.0010	0.3923	0.024*	
N2	0.54638 (17)	0.23011 (10)	0.02105 (8)	0.02622 (19)	
N3	0.43091 (14)	0.26487 (9)	0.47905 (7)	0.01813 (16)	
N4	0.41836 (15)	0.27045 (9)	0.58791 (7)	0.01990 (17)	
H5	0.5115	0.2250	0.6195	0.024*	
O1	0.75650 (12)	0.09474 (8)	0.56864 (6)	0.02078 (15)	
O2	0.36881 (18)	0.28657 (11)	0.03076 (8)	0.0393 (2)	
O3	0.64526 (18)	0.20633 (12)	−0.06636 (8)	0.0416 (2)	

### Atomic displacement parameters (Å^2^)

**Table:**

	*U*^11^	*U*^22^	*U*^33^	*U*^12^	*U*^13^	*U*^23^
C1	0.0163 (4)	0.0189 (4)	0.0187 (4)	0.0066 (3)	0.0039 (3)	0.0081 (3)
C2	0.0171 (4)	0.0202 (4)	0.0165 (4)	0.0077 (3)	0.0050 (3)	0.0084 (3)
C3	0.0160 (4)	0.0198 (4)	0.0176 (4)	0.0072 (3)	0.0050 (3)	0.0081 (3)
C4	0.0187 (4)	0.0222 (4)	0.0182 (4)	0.0089 (3)	0.0055 (3)	0.0091 (3)
C5	0.0229 (5)	0.0257 (4)	0.0165 (4)	0.0093 (4)	0.0049 (3)	0.0103 (3)
C6	0.0278 (5)	0.0344 (5)	0.0206 (4)	0.0158 (4)	0.0107 (4)	0.0125 (4)
C7	0.0242 (5)	0.0326 (5)	0.0217 (4)	0.0162 (4)	0.0100 (4)	0.0121 (4)
C8	0.0183 (4)	0.0217 (4)	0.0179 (4)	0.0084 (3)	0.0047 (3)	0.0085 (3)
C9	0.0181 (4)	0.0201 (4)	0.0169 (4)	0.0075 (3)	0.0053 (3)	0.0071 (3)
C10	0.0269 (5)	0.0326 (5)	0.0187 (4)	0.0138 (4)	0.0066 (4)	0.0120 (4)
C11	0.0357 (6)	0.0389 (6)	0.0195 (4)	0.0163 (5)	0.0127 (4)	0.0117 (4)
C12	0.0293 (5)	0.0329 (5)	0.0256 (5)	0.0152 (4)	0.0133 (4)	0.0085 (4)
C13	0.0250 (5)	0.0260 (4)	0.0274 (5)	0.0138 (4)	0.0085 (4)	0.0107 (4)
C14	0.0235 (5)	0.0236 (4)	0.0208 (4)	0.0112 (3)	0.0076 (3)	0.0111 (3)
N1	0.0203 (4)	0.0257 (4)	0.0196 (4)	0.0133 (3)	0.0068 (3)	0.0111 (3)
N2	0.0310 (5)	0.0332 (4)	0.0206 (4)	0.0144 (4)	0.0074 (3)	0.0136 (3)
N3	0.0184 (4)	0.0215 (3)	0.0171 (3)	0.0076 (3)	0.0056 (3)	0.0085 (3)
N4	0.0216 (4)	0.0265 (4)	0.0172 (3)	0.0130 (3)	0.0069 (3)	0.0108 (3)
O1	0.0218 (3)	0.0259 (3)	0.0201 (3)	0.0108 (3)	0.0059 (3)	0.0122 (3)
O2	0.0468 (5)	0.0577 (6)	0.0299 (4)	0.0370 (5)	0.0134 (4)	0.0241 (4)
O3	0.0475 (5)	0.0687 (6)	0.0280 (4)	0.0299 (5)	0.0198 (4)	0.0305 (4)

### Geometric parameters (Å, º)

**Table:**

C1—O1	1.2421 (11)	C9—C14	1.3942 (12)
C1—N1	1.3669 (11)	C9—N4	1.4029 (11)
C1—C2	1.4848 (12)	C10—C11	1.3845 (14)
C2—N3	1.3119 (11)	C10—H6	0.9500
C2—C3	1.4490 (12)	C11—C12	1.3913 (16)
C3—C4	1.3882 (12)	C11—H7	0.9500
C3—C8	1.4144 (12)	C12—C13	1.3863 (15)
C4—C5	1.3900 (13)	C12—H8	0.9500
C4—H2	0.9500	C13—C14	1.3919 (13)
C5—C6	1.3923 (13)	C13—H9	0.9500
C5—N2	1.4631 (12)	C14—H10	0.9500
C6—C7	1.3902 (13)	N1—H1	0.8800
C6—H3	0.9500	N2—O2	1.2267 (12)
C7—C8	1.3838 (13)	N2—O3	1.2316 (12)
C7—H4	0.9500	N3—N4	1.3202 (11)
C8—N1	1.3915 (11)	N4—H5	0.8800
C9—C10	1.3939 (13)		
			
O1—C1—N1	126.00 (8)	C14—C9—N4	121.93 (8)
O1—C1—C2	127.42 (8)	C11—C10—C9	119.56 (9)
N1—C1—C2	106.58 (7)	C11—C10—H6	120.2
N3—C2—C3	126.40 (8)	C9—C10—H6	120.2
N3—C2—C1	126.92 (8)	C10—C11—C12	120.53 (9)
C3—C2—C1	106.67 (7)	C10—C11—H7	119.7
C4—C3—C8	119.72 (8)	C12—C11—H7	119.7
C4—C3—C2	134.01 (8)	C13—C12—C11	119.47 (9)
C8—C3—C2	106.26 (7)	C13—C12—H8	120.3
C3—C4—C5	116.83 (8)	C11—C12—H8	120.3
C3—C4—H2	121.6	C12—C13—C14	120.93 (9)
C5—C4—H2	121.6	C12—C13—H9	119.5
C4—C5—C6	123.62 (9)	C14—C13—H9	119.5
C4—C5—N2	118.74 (8)	C13—C14—C9	118.93 (9)
C6—C5—N2	117.64 (8)	C13—C14—H10	120.5
C7—C6—C5	119.64 (9)	C9—C14—H10	120.5
C7—C6—H3	120.2	C1—N1—C8	110.92 (7)
C5—C6—H3	120.2	C1—N1—H1	124.5
C8—C7—C6	117.46 (9)	C8—N1—H1	124.5
C8—C7—H4	121.3	O2—N2—O3	123.29 (9)
C6—C7—H4	121.3	O2—N2—C5	118.18 (8)
C7—C8—N1	127.81 (8)	O3—N2—C5	118.50 (9)
C7—C8—C3	122.66 (8)	C2—N3—N4	116.98 (8)
N1—C8—C3	109.53 (8)	N3—N4—C9	121.85 (8)
C10—C9—C14	120.56 (9)	N3—N4—H5	119.1
C10—C9—N4	117.49 (8)	C9—N4—H5	119.1
			
O1—C1—C2—N3	2.70 (16)	C14—C9—C10—C11	1.64 (16)
N1—C1—C2—N3	−177.67 (9)	N4—C9—C10—C11	−176.92 (9)
O1—C1—C2—C3	−178.71 (9)	C9—C10—C11—C12	−0.59 (17)
N1—C1—C2—C3	0.91 (10)	C10—C11—C12—C13	−0.70 (18)
N3—C2—C3—C4	−2.73 (17)	C11—C12—C13—C14	0.97 (17)
C1—C2—C3—C4	178.67 (10)	C12—C13—C14—C9	0.06 (16)
N3—C2—C3—C8	176.72 (9)	C10—C9—C14—C13	−1.37 (15)
C1—C2—C3—C8	−1.87 (10)	N4—C9—C14—C13	177.12 (9)
C8—C3—C4—C5	−1.17 (14)	O1—C1—N1—C8	−179.93 (9)
C2—C3—C4—C5	178.23 (10)	C2—C1—N1—C8	0.44 (10)
C3—C4—C5—C6	−1.17 (15)	C7—C8—N1—C1	177.81 (10)
C3—C4—C5—N2	179.09 (8)	C3—C8—N1—C1	−1.68 (11)
C4—C5—C6—C7	2.57 (17)	C4—C5—N2—O2	−5.61 (15)
N2—C5—C6—C7	−177.69 (9)	C6—C5—N2—O2	174.63 (10)
C5—C6—C7—C8	−1.48 (16)	C4—C5—N2—O3	175.99 (10)
C6—C7—C8—N1	179.74 (10)	C6—C5—N2—O3	−3.77 (15)
C6—C7—C8—C3	−0.83 (16)	C3—C2—N3—N4	−177.84 (8)
C4—C3—C8—C7	2.21 (15)	C1—C2—N3—N4	0.47 (14)
C2—C3—C8—C7	−177.34 (9)	C2—N3—N4—C9	−179.85 (8)
C4—C3—C8—N1	−178.26 (8)	C10—C9—N4—N3	177.74 (9)
C2—C3—C8—N1	2.18 (10)	C14—C9—N4—N3	−0.80 (15)

### Hydrogen-bond geometry (Å, º)

**Table:**

*D*—H···*A*	*D*—H	H···*A*	*D*···*A*	*D*—H···*A*
N4—H5···O1	0.88	2.03	2.7479 (10)	137
N1—H1···O1^i^	0.88	1.96	2.8310 (10)	171
C10—H6···O3^ii^	0.95	2.63	3.5542 (13)	166
C12—H8···O2^iii^	0.95	2.47	3.3943 (13)	163

Symmetry codes: (i) −*x*+2, −*y*, −*z*+1; (ii) *x*, *y*, *z*+1; (iii) −*x*, −*y*+1, −*z*+1.
