# Supplementary material for: Crystal structure of (3E)-5-nitro-3-(2-phenyl­hydrazinyl­idene)-1H-indol-2(3H)-one
Source: Acta Crystallogr E Crystallogr Commun. 2017 Jan 13;73(Pt 2):168–72. doi: 10.1107/S2056989016020375 (PMC5290558; doi:10.1107/S2056989016020375)

SwissTargetPrediction Report for 5-nitroisatin-3-phenylhydrazone

SwissTargetPrediction report:

Reference:
Gfeller D., Michielin O. & Zoete V.
Shaping the interaction landscape of bioactive molecules, *Bioinformatics* (2013) 29:3073-3079.

Query Molecule

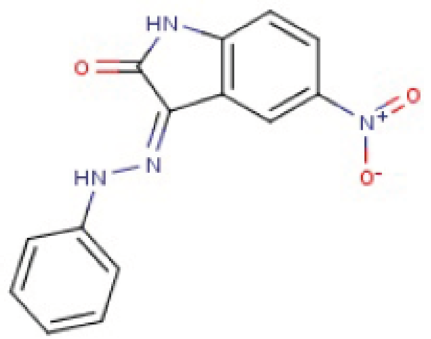

Frequency of Target Class

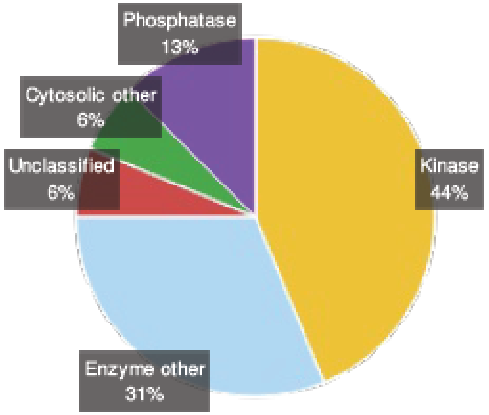

Supplement: Supplementary file 3 [file e-73-00168-sup3.pdf]
